# Predominance of Escherichia-Shigella in Gut Microbiome and Its Potential Correlation with Elevated Level of Plasma Tumor Necrosis Factor Alpha in Patients with Tuberculous Meningitis

**DOI:** 10.1128/spectrum.01926-22

**Published:** 2022-11-09

**Authors:** Shanshan Li, Jidong Guo, Rongmei Liu, Fuzhen Zhang, Shu’an Wen, Yi Liu, Weicong Ren, Xuxia Zhang, Yuanyuan Shang, Mengqiu Gao, Jie Lu, Yu Pang

**Affiliations:** a Department of Bacteriology and Immunology, Beijing Chest Hospital, Capital Medical University/Beijing Tuberculosis & Thoracic Tumor Research Institute, Beijing, People’s Republic of China; b Department of Tuberculosis, Beijing Chest Hospital, Capital Medical University/Beijing Tuberculosis & Thoracic Tumor Research Institute, Beijing, People’s Republic of China; c Department of Infectious Diseases, Beijing Friendship Hospital, Capital Medical University, Beijing, People’s Republic of China; d Beijing Key Laboratory for Pediatric Diseases of Otolaryngology, Head and Neck Surgery, MOE Key Laboratory of Major Diseases in Children, Beijing Pediatric Research Institute, Beijing Children’s Hospital, Capital Medical University, National Center for Children’s Health, Beijing, People’s Republic of China; Johns Hopkins University School of Medicine

**Keywords:** tuberculous meningitis, gut microbiota, *Escherichia-Shigella*, TNF-α, claudin-5

## Abstract

Tuberculous meningitis (TBM), the most lethal and disabling form of tuberculosis (TB), may be related to gut microbiota composition, warranting further study. Here we systematically compared gut microbiota compositions and blood cytokine profiles of TBM patients, pulmonary TB patients, and healthy controls. Notably, the significant gut microbiota dysbiosis observed in TBM patients was associated with markedly high proportions of Escherichia-Shigella species as well as increased blood levels of tumor necrosis factor alpha (TNF-α) and interleukin 6 (IL-6). Next, we obtained a fecal bacterial isolate from a TBM patient and administered it via oral gavage to mice in order to develop a murine gut microbiota dysbiosis model for use in exploring mechanisms underlying the observed relationship between gut microbial dysbiosis and TBM. Thereafter, cells of commensal Escherichia coli (*E. coli*) were isolated and administered to model mice by gavage and then mice were inoculated with Mycobacterium tuberculosis (*M. tuberculosis*). Subsequently, these mice exhibited increased blood TNF-α levels accompanied by downregulated expression of tight junction protein claudin-5, increased brain tissue bacterial burden, and elevated central nervous system inflammation relative to corresponding indicators in controls administered PBS by gavage. Thus, our results demonstrated that a signature dysbiotic gut microbiome profile containing a high proportion of E. coli was potentially associated with an increased circulating TNF-α level in TBM patients. Collectively, these results suggest that modulation of dysbiotic gut microbiota holds promise as a new strategy for preventing or alleviating TBM.

**IMPORTANCE** As the most severe form of tuberculosis, the pathogenesis of tuberculous meningitis (TBM) is still unclear. Gut microbiota dysbiosis plays an important role in a variety of central nervous system diseases. However, the relationship between gut microbiota and TBM has not been identified. In our study, significant dysbiosis in gut microbiota composition with a high proportion of E. coli and increased levels of TNF-α in plasma was noted in TBM patients. A commensal E. coli was isolated and shown to increase the plasma level of TNF-α and downregulate brain tight junction protein claudin-5 in the murine model. Gavage administration of E. coli aggravated the bacterial burden and increased the inflammatory responses in the central nervous system after M. tuberculosis infection. Dysbiosis of gut microbiota may be a promising therapeutic target and biomarker for TBM prevention or treatment.

## INTRODUCTION

Tuberculosis (TB) remains a global public health concern, with an estimated 9.9 million TB cases and 1.3 million deaths from TB reported in 2020 (1, 2). Although most TB infections affect the lungs and are thus referred to as pulmonary TB (PTB) cases, TB bacilli can also invade almost any other organ to cause extrapulmonary TB (EPTB) (3, 4). Central nervous system tuberculosis (CNS-TB) is the most lethal and disabling form of EPTB, of which tuberculous meningitis (TBM) is the most severe manifestation (5). Importantly, TBM is almost always underdiagnosed, due to a lack of effective microbiological tests (6). Nevertheless, more than 100,000 new cases have been estimated to occur each year (5, 7) in spite of TBM diagnostic challenges stemming from the paucibacillary nature of the disease. Such challenges have prompted researchers to search for alternative diagnostic biomarkers for use in achieving early detection of TBM cases (8, 9). Unfortunately, the lack of knowledge with regard to TBM immunopathology has greatly hindered efforts to develop effective host-directed therapies and diagnostic tools.

The gut microbiota plays essential roles in the functions of many organs in the body, including the brain (10, 11). Intriguingly, results of numerous preclinical and clinical studies have highlighted interactions that occur between the CNS and the gastrointestinal system (12, 13). Mechanistically, such interactions may involve the entry of microbially derived metabolites into the circulation, tissues, or organs, where they may stimulate host immune cells to secrete cytokines that affect blood-brain barrier (BBB) permeability (14). Indeed, results of previous studies have shown that increased BBB permeability is undoubtedly a risk factor for CNS infections caused by circulating bacterial pathogens. For example, results of a murine model-based study demonstrated that exposure of germfree mice to pathogen-free gut microbiota decreased BBB permeability through the upregulation of BBB tight junction proteins (15), thus highlighting the fact that intestinal homeostasis is highly important for healthy gut-brain communication.

Experimental characterizations of gut microbiomes of TB patients and M. tuberculosis-infected animal models that revealed significantly decreased bacterial diversity in gut microbiomes of PTB patients have been reported (16, 17), while also reporting gut microbiota profiles of various TB patient populations and animal models (17). For example, relative abundances of *Ruminococcaceae* and/or *Lachnospiraceae*, two of the most abundant bacterial families detected in healthy human colon mucosa, were significantly altered after M. tuberculosis infection (18, 19). Moreover, antibiotic therapy influenced both pathogen killing and microbiome-driven immunomodulation (20). However, few reported TB patient studies have demonstrated direct or indirect impacts of gut microbiomes on host immune functions at distant sites (21).

TBM pathogenesis is believed to begin with the spread of tubercule bacilli in the lungs to the BBB via lymphohematogenous dissemination, followed by bacterial traversal of the BBB and entry into the brain (7). In view of the close connection between the host CNS and intestine, it would be interesting to explore whether altered gut microbiota contributes to TBM CNS pathogenesis, an association that has not yet been clearly elucidated to our knowledge. In this study, we performed a comparative analysis of the gut microbiota of TBM patients, pulmonary TB patients, and healthy controls in order to identify TBM-associated gut microbiota markers. In addition, we generated a dysbiosis mouse model for use in investigating potential mechanisms whereby gut microbiome dysbiosis supports TBM pathogenesis.

## RESULTS

### General characteristics of gut microbiota in TBM patients.

**FIG 1:**
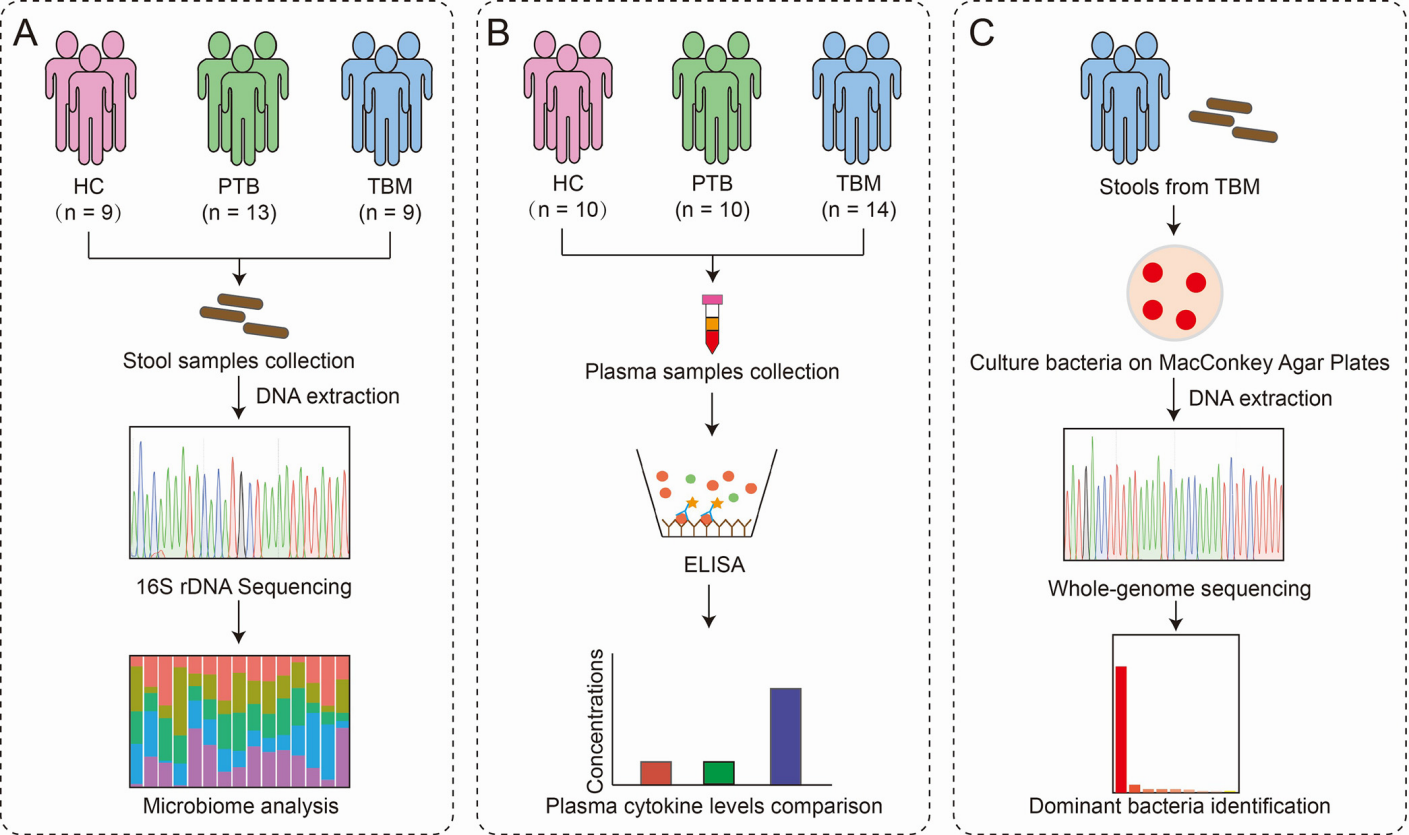
Overview of the experimental workflow for human samples. (A) Gut microbiome analysis using fecal samples from the HC, PTB, and TBM groups. (B) Calculation of cytokine expression levels in plasma samples from the HC, PTB, and TBM groups. (C) Identification of dominant gut bacteria using whole-genome sequencing. HC, healthy control; PTB, patients with pulmonary tuberculosis; TBM, patients with tuberculous meningitis.

A workflow for human samples is depicted in Fig. 1. A total of 31 participants, including 9 healthy controls (HCs), 13 hospitalized PTB patients, and 9 hospitalized TBM patients, were recruited for gut microbiota analysis. As shown in Table 1, no significant differences were observed with regard to participant age, sex, and body mass index (BMI) characteristics among the three groups. Overall, a total of 4,285,213 (4.3 million) high-quality reads with an average length of 466 bp were obtained from 31 fecal samples. The average numbers of reads obtained for HC, PTB, and TBM groups were 162,594, 130,590, and 124,911, respectively.

**TABLE 1:** Demographic features for TBM and PTB patients and healthy study subjects

Characteristic	Value for indicate group^*a*^			Significance (*P*)^*c*^
	HC (*n* = 9)	PTB (*n* = 13)	TBM (*n* = 9)	
Age (yr)	30 (26, 35)	34 (19, 52)	29 (19, 41)	0.7
No. of males/no. of females	6/3	10/3	6/3	0.39
BMI^*b*^	23.62 ± 0.99	21.3 ± 0.71	21.93 ± 1.15	0.21

a Measurement data based on a normal distribution are presented as the mean ± standard deviation, while data not based on a normal distribution are expressed as median values based on interquartile ranges. HC, healthy control; PTB, patients with pulmonary tuberculosis; TBM, patients with tuberculous meningitis.

b BMI, body mass index.

c Significance level, *P *< 0.05.

**FIG 2:**
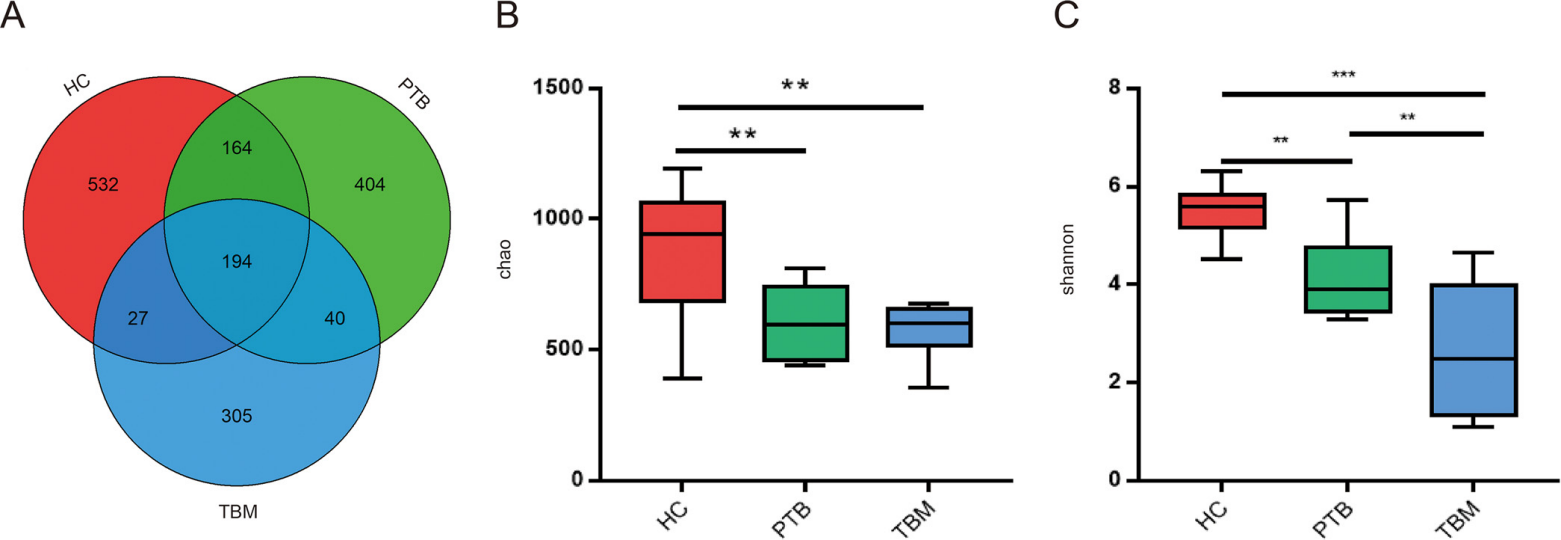
Gut microbial diversity differences among TBM patients, PTB patients, and healthy individuals. (A) Venn diagram showing shared and unique operational taxonomic units (OTUs) identified in fecal samples from the HC, TB, and TBM groups. (B and C) α-Diversity was assessed using Chao (B) and Shannon (C) indexes. Results are shown as the mean ± SEM as determined using the Kruskal-Wallis test. ****, *P < *0.0001; **, *P < *0.01. HC, healthy control; PTB, patients with pulmonary tuberculosis; TBM, patients with tuberculous meningitis.

Operational taxonomic unit (OTU) clustering analysis of reads yielded 1,666 OTUs (Fig. 2A). Notably, the total number of OTUs obtained for the HC group (*n *= 917) was much higher than that obtained for the PTB group (*n *= 802) and the TBM group (*n *= 566). Moreover, average OTU numbers for TBM and PTB groups were significantly lower than the average OTU number of the HC group (418 ± 61 and 472 ± 59 versus 674 ± 146, respectively, *P < *0.0001). In addition, consistently significant differences in α-diversity index values were noted among the three groups, with the most prominent difference observed between the HC and TBM groups (Fig. 2B and C). Taken together, these results revealed that the microbial diversity of the gut microbiota of TBM patients was lesser than that of healthy control subjects.

### Alterations of gut microbiota between TBM and control groups.

**FIG 3:**
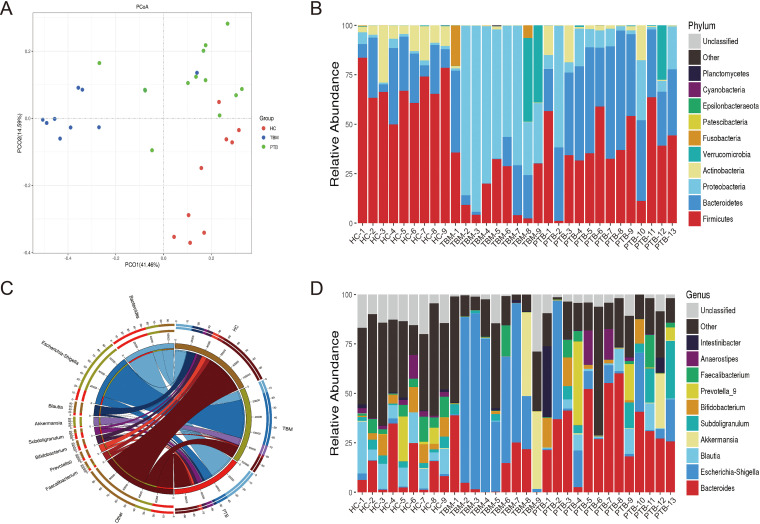
Gut microbiota composition of TBM patients, PTB patients, and healthy subjects. (A) Principal-coordinate analysis (PCoA) based on weighted UniFrac distance analysis showing PCoA distributions along the principal-component (PCO) axis for the three subject groups. Percentages marked on the horizontal and vertical axes are the contributions of the principal coordinate to data differences for the sample matrix. (B) Stack diagram of relative phylum-level gut microbiota abundances in the HC, PTB, and TBM groups. (C) Circos plot showing genus-level groupings, whereby the top 10 genera in all samples/groups with tag numbers of >2,000 are shown in the Circos plot. (D) Genus-level stack diagram showing relative abundances of gut microbiota genera in the HC, PTB, and TBM groups. HC, healthy control; PTB, patients with pulmonary tuberculosis; TBM, patients with tuberculous meningitis.

Results of principal-coordinate analysis (PCoA) of weighted UniFrac distance data revealed that the OTUs of gut microbiota of the HC, PTB, and TBM groups fell into distinct clusters, and a permutational multivariate analysis of variance (PERMANOVA) test showed that the bacterial community structure was significantly different (*P = *0.001) among these three groups (see Table S2 in the supplemental material), thus indicating that marked microbiome compositional differences existed across the three groups (Fig. 3A). Based on these results, we subsequently compared baseline gut microbiota compositions of the participants of all three groups.

At the phylum level (Fig. 3B), a total of 9 bacterial phyla were detected in HC fecal samples, including the dominant phyla *Firmicutes* (68.15%), *Bacteroidetes* (18.76%), and *Actinobacteria* (10.51%), based on total sequence numbers. For the PTB group, a total of 9 bacterial phyla were detected in fecal samples, including *Firmicutes* (38.28%), *Bacteroidetes* (37.73%), and *Actinobacteria* (12.50%) as the top three dominant phyla. For the TBM group, 10 bacterial phyla were classified, including *Proteobacteria* (51.45%), *Firmicutes* (17.72%), and *Bacteroidetes* (11.77%) as the top three dominant phyla. Notably, there were significant differences in microbiota between TBM and HC individuals: the microbiota of TBM patients exhibited dysbiosis, which was detected based on a relatively greater proportion of *Proteobacteria* (*P < *0.001) and relatively lower proportions of *Firmicutes* (*P < *0.01) and *Actinobacteria* (*P < *0.01) than those of the microbiota of the HC group (Fig. S1A). Meanwhile, relatively larger proportions of *Proteobacteria* (*P < *0.05) and *Bacteroidetes* (*P < *0.001) and a relatively lower proportion of *Firmicutes* (*P < *0.001) were observed in PTB group microbiota (Fig. S1B) than in the HC group microbiota. Moreover, a significantly greater proportion of *Proteobacteria* (*P < *0.001) and significantly lower proportions of *Firmicutes* (*P < *0.001), *Bacteroidetes* (*P < *0.001), and *Actinobacteria* (*P < *0.05) were observed in TBM group microbiota than in the PTB group microbiota (Fig. S1C).

At the genus level (Fig. 3C and D), Escherichia-Shigella accounted for nearly half of the genera detected in high-throughput sequencing reads of microbiota of TBM fecal samples (48.72%), a significantly greater proportion than that obtained for samples from the PTB (12.11%, *P < *0.05) and HC (1.04%, *P < *0.01) groups, thus indicating that this dysbiosis characteristic was exclusively associated with TBM. Conversely, proportions of *Blautia* (0.14% versus 11.42%, *P < *0.001), *Bifidobacterium* (0.27% versus 5.99%, *P < *0.001), *Fusicatenibacter* (0.04% versus 2.90%, *P < *0.05), and *Anaerostipes* (0.04% versus 2.83%, *P < *0.05) in TBM samples were significantly lower than those (within the top 10 dominant genera) in HC fecal samples. Meanwhile, comparisons of TBM and PTB fecal microbiota compositions revealed that the relative proportions of *Bacteroides* (11.92% versus 33.77%, *P < *0.01), *Blautia* (0.14% versus 4.16%, *P < *0.05), and CAG-56 (0.0015% versus 0.0089%, *P < *0.05) in TBM fecal samples were significantly lower than those in PTB fecal samples. Results corresponding to genus-level differences, which were evaluated for significance using Welch’s *t* test, are shown for the TBM versus HC (Fig. S2A), PTB versus HC (Fig. S2B), and TBM versus PTB (Fig. S2C) groups.

### Identification of dominant intestinal species of Escherichia-Shigella in TBM patients.

To identify the predominant Escherichia-Shigella species in fecal samples from TBM patients, we conducted a culture-based analysis of intestinal Escherichia-Shigella species by culturing bacteria on MacConkey agar plates. Only red colonies were observed on MacConkey agar plates, a result that suggested that all isolates were E. coli. Further analysis of bacteria from the red colonies via whole-genome sequencing (WGS) revealed that sequences of nearly 90% of the reads matched the E. coli sequence, thus confirming that E. coli was the dominant bacterial species detected in TBM patient microbiota (Fig. S3). A clinical E. coli isolate (CEC) from a male TBM patient with a dominant proportion of the genera Escherichia-Shigella in stool sample was identified as belonging to serotype O48:H28.

### Altered cytokine responses in TBM patients.

**FIG 4:**
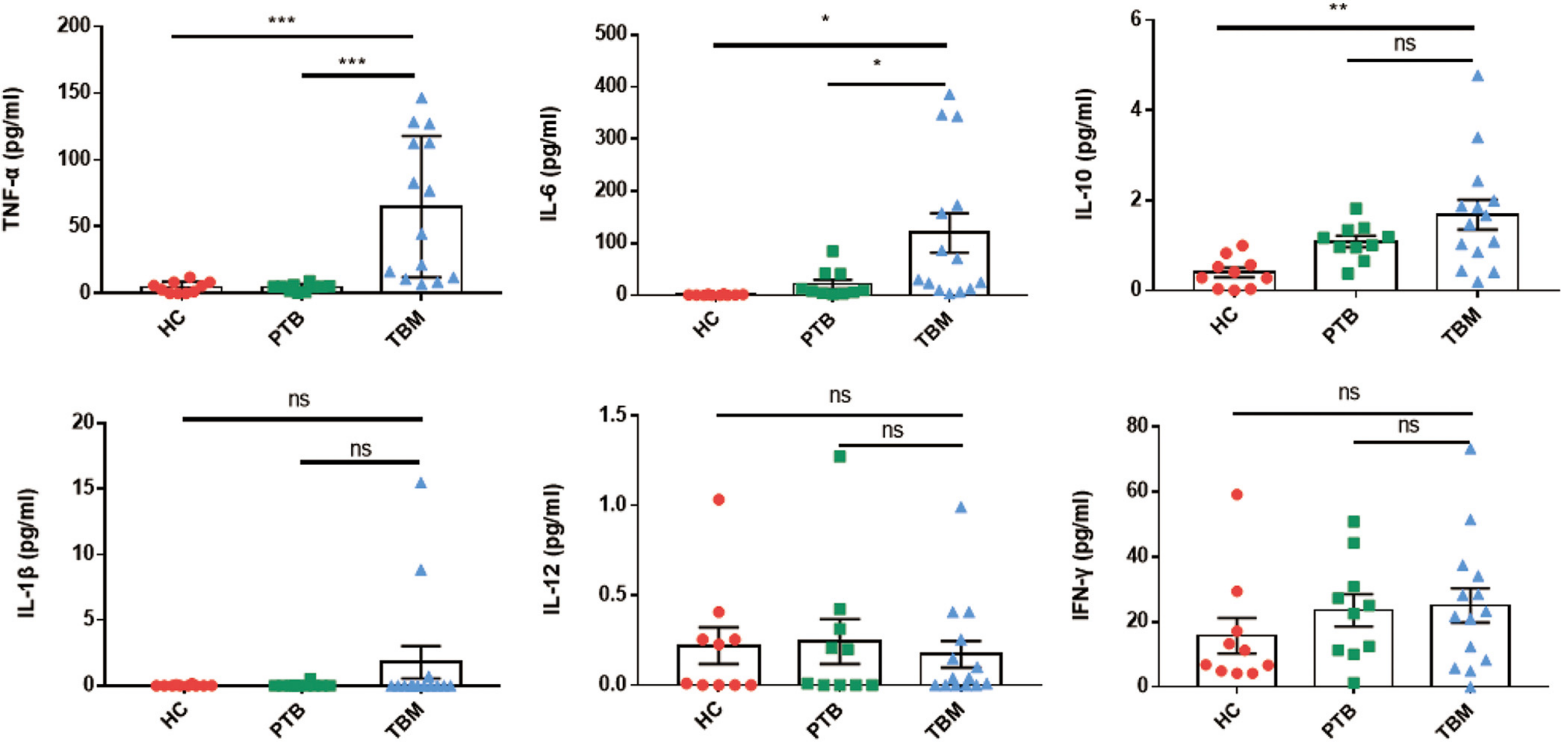
Plasma cytokine levels in TBM and PTB patients and healthy subjects. Expression levels of TNF-α, IL-6, IL-10, IL-1β, IL-12, and IFN-γ in plasma samples from HC individuals and PTB and TBM patients. Each result is shown as the mean ± SEM as determined based on analysis using the Kruskal-Wallis test (ns, not significant; ***, *P < *0.001; **, *P < *0.01; *, *P < *0.05). HC, healthy control; PTB, patients with pulmonary tuberculosis; TBM, patients with tuberculous meningitis.

Due to the fact that gut microbiome dysbiosis is often accompanied by abnormal production of proinflammatory cytokines (22, 23), here we investigated whether plasma concentrations of proinflammatory cytokines of the TBM patient group differed significantly from the corresponding concentrations for the other two groups (Fig. 4). Based on analysis of the levels of six cytokines in plasma samples obtained from 14 hospitalized TBM patients, 10 hospitalized PTB patients, and 10 healthy individuals, the plasma levels of TNF-α (64.9 ± 53.0 pg/mL versus 4.5 ± 4.3 pg/mL, *P < *0.001), IL-6 (119.8 ± 140.5 pg/mL versus 1.0 ± 1.0 pg/mL, *P < *0.01), and IL-10 (1.7 ± 1.2 pg/mL versus 0.4 ± 0.3 pg/mL, *P < *0.01) were found to be markedly increased in the TBM group compared to the HC group. Meanwhile, comparisons of plasma cytokine levels between TBM and PTB groups revealed significantly higher levels of TNF-α (64.9 ± 53.0 pg/mL versus 4.4 ± 2.6 pg/mL, *P < *0.001) and IL-6 (119.8 ± 140.5 pg/mL versus 21.4 ± 26.8 pg/mL, *P < *0.05) in the TBM group. Higher levels of TNF-α and IL-6 in the TBM group could be due to several reasons, such as M. tuberculosis circulating in their blood; whether there was a correlation between gut microbiome dysbiosis and altered cytokine responses in TBM patients needed further evidence.

### Increased plasma TNF-α level and BBB permeability in the mouse model of gut microbiome dysbiosis without M. tuberculosis infection.

Previous studies showed that a single oral gavage with live E. coli could increase plasma TNF-α in mice (24) and that intravenous administration of TNF-α caused an increase in BBB permeability (25). Considering that a CEC in TBM patients was identified as E. coli O48: H28, it was possible that the CEC might elevate the plasma TNF-α level, leading to increased BBB permeability. Therefore, we first evaluated the impact of the CEC on the plasma TNF-α level and BBB permeability by using a mouse model of gut microbiome dysbiosis without M. tuberculosis infection.

**FIG 5:**
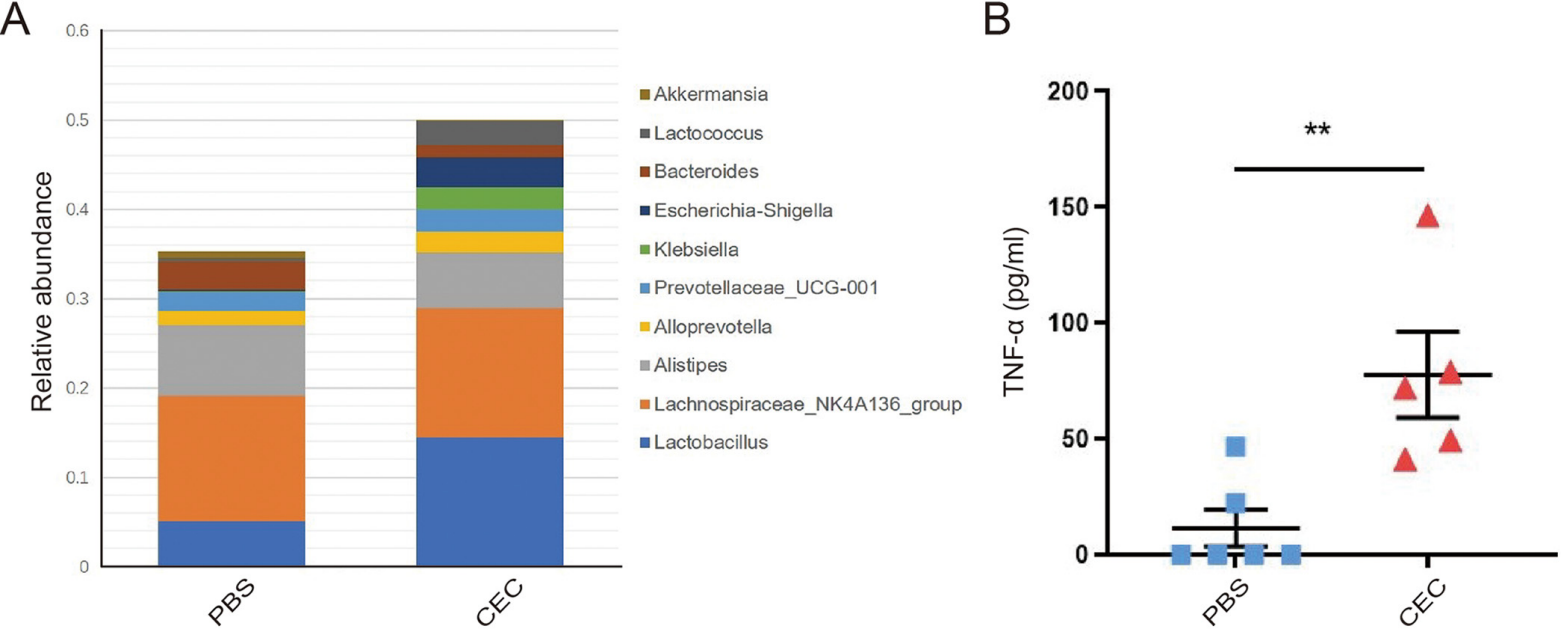
Gut microbiome dysbiosis increased the level of circulating TNF-α in a mouse model without M. tuberculosis infection. (A) Stack diagram of relative abundances of gut microbiota genera in mouse stools after 2 weeks of PBS or CEC administered by gavage. (B) TNF-α levels in serum from C57BL/6 mice administered PBS or CEC by gavage. Each result is shown as the mean ± SEM as determined based on analysis conducted using the Kruskal-Wallis test (**, *P < *0.01). CEC, clinical E. coli isolate.

The mouse model was generated through intragastric administration of CEC; mice receiving phosphate-buffered saline (PBS) by gavage served as controls (Fig. S4A). After a 14-day administration by gavage, mice receiving CEC by gavage exhibited lower growth rates (based on body weight) than mice in the control groups (Fig. S5A), while one mouse died after receiving CEC by gavage for 2 weeks. As shown in Fig. 5A, the relative abundance of bacteria belonging to the genera Escherichia-Shigella was substantially greater in the gut microbiota of CEC-treated mice than in the microbiota of control group mice.

We then detected the expression levels of plasma cytokines (TNF-α and IL-6) and biomarkers of BBB permeability (claudin-5, zonula occludens-1 [ZO-1], and occludin) in mice with altered microbiota. As shown in Fig. 5B, higher concentrations of circulating TNF-α were consistently and significantly observed in mice administered CEC by gavage than in those administered PBS by gavage (77.64 ± 41.46 pg/mL versus 11.47 ± 19.38 pg/mL, *P < *0.01). In contrast, no differences in plasma levels of IL-6 were observed among the two groups (Fig. S5B). As shown in Fig. S5C, mRNA expression levels of claudin-5 and ZO-1 decreased significantly after administration of CEC in comparison with the levels in control group mice. In contrast, no statistically significant intergroup differences in occludin mRNA expression levels were observed. These results suggest that the unique microbiota profile observed in TBM patients was associated with a markedly elevated proinflammatory TNF-α response, which might relate to increased BBB permeability.

### Gut microbiome dysbiosis correlates with increased M. tuberculosis burden in mouse brain tissues.

**FIG 6:**
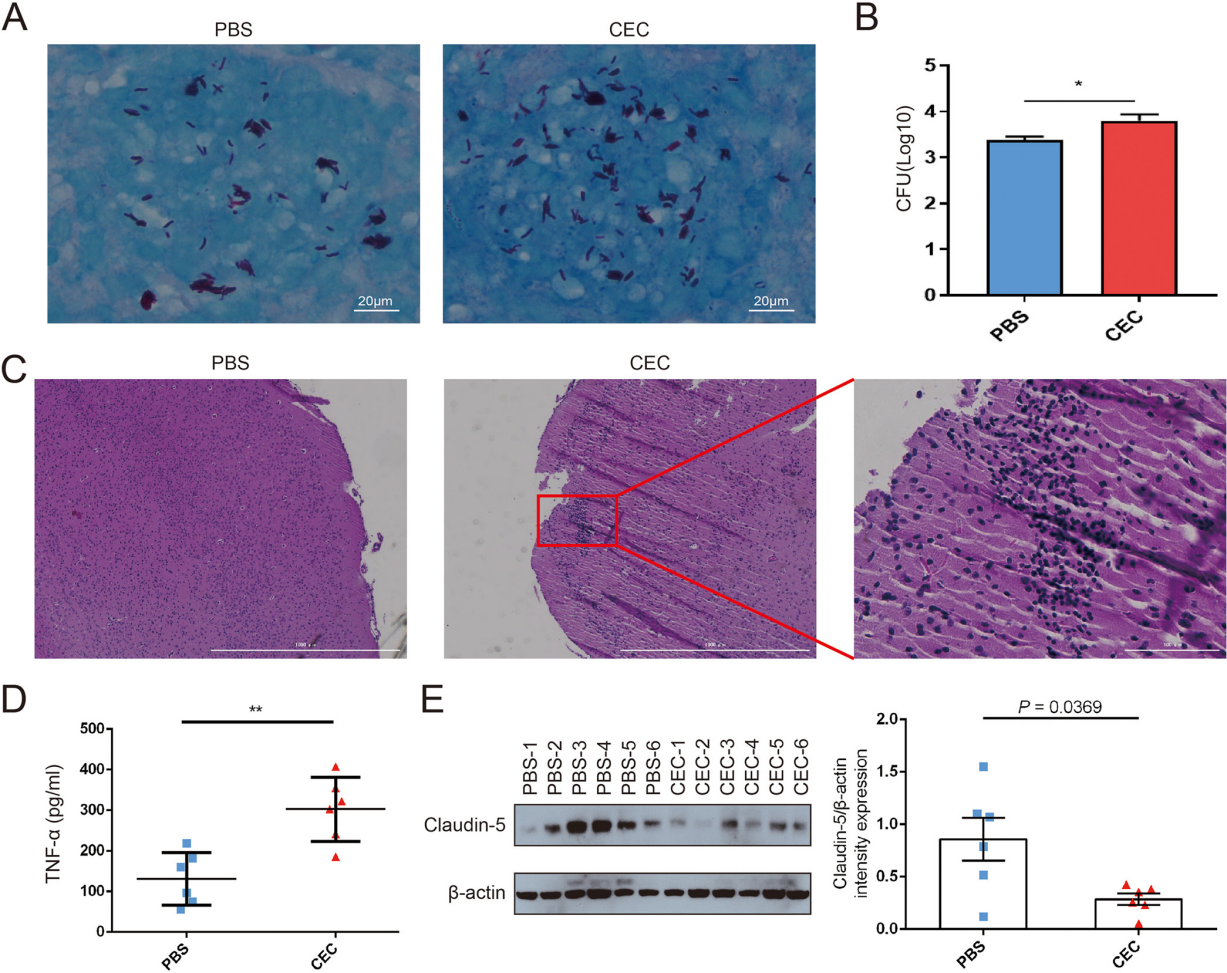
Gut microbiome dysbiosis was associated with increased M. tuberculosis burden in mouse brain tissues. (A) Acid-fast bacilli were visualized by ZN staining in paraffin wax sections of brain tissue from C57BL/6 mice administered PBS or CEC by gavage. (B) Brain bacterial counts 2 weeks after *M. tuberculous* injection of mice previously treated by gavage with CEC or PBS. (C) Inflammation of mouse brain tissues as determined from stained brain tissue sections via microscopy. (D) TNF-α levels in serum from C57BL/6 mice 2 weeks after *M. tuberculous* injection. (E) Left, Western blotting results showing claudin-5 levels in brain tissues of C57BL/6 mice; right, quantitative analysis of claudin-5 expression in brain tissues of C57BL/6 mice. Data were normalized based on β-actin expression as the loading control and are expressed as fold changes. Each value is expressed as the mean ± SEM as determined using Student’s *t* test for the analysis, with a *P *of <0.05 (*) considered significant. CEC, clinical E. coli isolate.

To further investigate the influence of gut microbiome dysbiosis on bacterial burden in brain tissues, M. tuberculosis H37Rv (ATCC 27294) was administered to C57BL/6 mice via intravenous tail vein injection after the mice had received CEC or PBS by gavage for 2 weeks (Fig. S4B). Bacterial loads in blood at day 1 after M. tuberculosis infection were evaluated (Fig. S5D), and successful infection in brain tissues at day 14 were confirmed by Ziehl-Neelsen (ZN) staining (Fig. 6A). The numbers of CFU in M. tuberculosis cultures prepared from mouse brain homogenates were determined. The results revealed that CFU numbers detected in brain tissues of M. tuberculosis-infected mice administered CEC by gavage were significantly higher than the corresponding numbers detected in brain tissues of M. tuberculosis-infected mice administered PBS by gavage (Fig. 6B). In addition, hematoxylin and eosin (H&E) staining results revealed obvious leukocyte infiltration of brain tissues of mice administered CEC by gavage, suggesting that inflammation within brain tissues of the M. tuberculosis-infected CEC group mice was more severe than that observed in brains of PBS group mice (Fig. 6C). As shown in Fig. 6D, mice administered CEC by gavage had a significantly higher level of circulating TNF-α than those administered PBS by gavage (302.67 ± 32.38 pg/mL versus 131.40 ± 26.39 pg/mL, *P < *0.01). The average expression level of claudin-5 in brain tissues in the CEC gavage group was significantly higher than that in PBS gavage group (Fig. 6E).

## DISCUSSION

The results of this study demonstrated that gut microbiome dysbiosis in TBM patients was characterized by the predominance of Escherichia-Shigella, which possibly elevated the TNF-α level in plasma, suggesting a potential mechanism underlying TBM pathogenesis. Importantly, dysbiosis of gut microbiota in TBM patients was specifically associated with a relatively increased abundance of intestinal proinflammatory bacteria of the genera Escherichia-Shigella and a dramatic reduction in the relative proportions of beneficial bacteria belonging to the genera *Blautia*, *Bifidobacterium*, *Fusicatenibacter*, and *Anaerostipes*. Moreover, based on the results of experiments conducted using a mouse model of gut microbiota dysbiosis, we hypothesized that gut microbiome dysbiosis in TBM patients potentially upregulated plasma TNF-α, which might influence BBB permeability and M. tuberculosis infection in brain.

In this study, we identified bacterial genera that were significantly and specifically associated with TBM. Notably, a marked increase in the relative proportion of Escherichia-Shigella was observed in the gut microbiota of TBM patients. Interestingly, results of a previous study had indicated that expansion of *Proteobacteria*, the parent family of Escherichia-Shigella, may serve as a microbial signature of gut dysbiosis (26). Notably, this result aligned with results presented here that demonstrated that expansion of Escherichia-Shigella was associated with gut microbiota dysbiosis in TBM patients. Several studies have shown that disruption of intestinal homeostasis may lead to decreased intestinal barrier function and increased plasma levels of inflammatory cytokines (27–29). Here, we found that TBM patients exhibited significantly higher concentrations of plasma TNF-α than did PTB patients and healthy controls, and further animal experiments revealed that a clinical E. coli isolate administered by gavage in mice could increase the expression level of circulating TNF-α. Importantly, studies have shown that TNF-α can participate in BBB breakdown associated with other pathological disorders through downregulation of expression of tight junction proteins (30, 31), and intracerebral injection of TNF-α into mice led to dramatically elevated BBB permeability (25). In addition, injections of TNF-α into tissues have been shown to trigger NF-κB pathway signaling that led to substantially decreased expression of the tight junction proteins occludin, claudin-5, and ZO-1 (32, 33). Moreover, in a previous animal-based study, exposure of germfree mice to pathogen-free gut microbiota resulted in both decreased BBB permeability and upregulated expression of tight junction proteins, thus suggesting that altered gut microbiota is associated with BBB integrity (15). Therefore, gut microbiota dysbiosis in TBM patients might lead to impaired BBB integrity and increased BBB permeability as a possible pathogenic mechanism underlying the occurrence of TBM. Accordingly, in the present study, we also observed decreased expression of claudin-5 and ZO-1 in mice administered CEC by gavage. Taken together, our results and the results of previous studies collectively suggest that circulating TNF-α might serve as a bridge between intestinal dysbiosis and CNS infection.

Although the exact mechanisms underlying expansions of Escherichia-Shigella in the gut microbiota of TBM individuals remain unclear, our findings provide important clues toward improving clinical management of TBM patients. On the one hand, elevated proportions of Escherichia-Shigella in the gut microbiota of these patients could serve as a diagnostic marker of TBM disease, warranting additional clinical investigations to evaluate the predictive value of this marker when used for this purpose. On the other hand, opportunistic bacteria appear to have greater resistance to antibiotics than beneficial bacteria, such that antibiotic treatment would thereby drive expansion of opportunistic organisms within the gut-associated microbial community (34). Considering the fact that multiple agents are used for TB treatment, the question must be raised as to whether the expected catastrophic shift in microbiota after antibiotic treatment would increase the risk of TBM occurrence in pulmonary TB patients and worsen the prognosis of TB patients with TBM. Taken together, the results presented here indicate that early correction of gut microbiota disturbances holds promise as a potential therapy for decreasing TBM-associated morbidity and mortality. Nevertheless, clinical trials are warranted to verify this hypothesis.

We acknowledge several limitations of the study. First, although we used the same diagnostic criteria for patient inclusion regardless of patient cohort, the use of different cohorts for gut microbiome and cytokine analyses may have statistically weakened our results pertaining to associations we found between cytokine abnormalities and gut microbiome changes. Second, multiple factors likely influence gut microbiota diversity and composition, such as antibiotic use, diet, and genetic background. Due to the small number of patients included in this study, statistically robust analysis of confounding factors using multivariate mathematic models could not be conducted, warranting additional investigations involving larger prospective cohorts of TBM patients and patients with other types of CNS meningitis in order to verify our results. Third, another concern is that these results could not rule out the confounding initial immune response by artificiality of single-species inoculation in the experimental mouse model. Further correction of bacterial dysbiosis by fecal transplantation would provide more evidence that greater bacterial diversity and a shift in microorganism species potentially correlate with a decreased level of proinflammatory TNF-α. Fourth, despite previous experimental evidence showing that TNF-α plays an important role in the BBB breakdown (15, 25, 32) and that anti-TNF-α treatment can abrogate BBB damage (35, 36), we did not validate the correlation of elevated TNF-α levels and barrier impairment in the mouse model. Further animal experiments by employment of TNF-α blockade and exogenous TNF-α will strengthen the basis for our conclusions. Finally, whole-genome sequencing was conducted for only one isolate, i.e., the isolate that was obtained from the TBM patient with the highest gut microbiota abundance of E. coli. Thus, the sequence obtained for this isolate should be confirmed by sequencing of additional TBM patient isolates in order to increase the clonal diversity of sequenced E. coli strains and to test the strength of our conclusion. Nevertheless, results of this study highlight the potential of intestinal microbiota compositional modulation as a promising new strategy for use in correcting gut microbiota dysbiosis to prevent or alleviate TBM.

In summary, here we showed that TBM gut microbiota profiles differed from those of healthy individuals and PTB patients. More specifically, the signature TBM dysbiotic gut microbiome profile found here was associated with a greater relative abundance of E. coli that, in turn, was associated with increased TNF-α levels in the blood of TBM patients. However, additional studies are needed to elucidate which bacterial components play key roles in processes associated with increased circulating proinflammatory cytokine levels in order to establish whether correction of gut dysbiosis may prevent or alleviate CNS M. tuberculosis infections in TB patients.

## MATERIALS AND METHODS

### Ethics statement.

All study procedures using human samples were approved by the Ethics Committee of Beijing Chest Hospital, which is affiliated with Capital Medical University (YJS-2020-009). Written informed consent was obtained from each individual. All animal procedures were approved by the Ethics Committee of the Experimental Animal Care of Beijing Chest Hospital, Capital Medical University (2021-016). All experimental protocols for the experiments on M. tuberculosis were approved by the Biosafety Committee of Beijing Chest Hospital and conducted in accordance with national biosafety guidelines in China.

### Participants.

For characterization of gut microbiomes, three groups of individuals, including healthy controls, PTB patients, and TBM patients, were enrolled from January to June 2019 at Beijing Chest Hospital, a national TB-designated hospital in China. General demographic characteristics of these individuals, such as sex, age, and body mass index (BMI), were compared in the study. An independent cohort composed of healthy controls, PTB patients, and TBM patients who were recruited at Beijing Chest Hospital from July to December 2019 was sampled to assess the expression levels of blood plasma cytokines. Pulmonary TB was diagnosed based on criteria that are outlined in the National Health Commission of the People's Republic of China pulmonary tuberculosis diagnosis and treatment guidelines (WS 288–2017; China), while TBM was diagnosed based on expert consensus (37). PTB patient inclusion criteria were as follows: (i) aged 18 to 70 years; (ii) positive M. tuberculosis detection results obtained for sputum smears and/or cultures and/or positive GeneXpert MTB/RIF results (Cepheid, USA); (iii) pulmonary imaging features indicative of TB. All PTB patients who were enrolled in the study met all three criteria. All enrolled TBM patients had been diagnosed with definite TBM, were aged 18 to 70 years, and tested positive for M. tuberculosis complex bacilli in cerebrospinal fluid (CSF) by GeneXpert MTB/RIF assay. Healthy controls were recruited after they had received routine annual physical examinations showing the absence of TB based on negative interferon gamma release assay (IGRA) results, absence of abnormal pulmonary findings, and absence of CNS disease symptoms. In addition, healthy subjects were selected based on the following inclusion criteria: (i) aged 18 to 70 years; (ii) healthy BMI and test results showing normal liver and kidney function, normal stools, and normal levels of fasting blood glucose, blood lipids, and urinary function indicators; and (iii) no history of pulmonary and brain diseases. Exclusion criteria were as follows: (i) prior treatment with systemic antituberculous therapy for more than 1 week; (ii) prior treatment with any steroid drugs; (iii) receipt of treatment with extensive antibiotic therapy for more than 1 week during the previous 6 months; (iv) comorbidities, such as diabetes, malignancy, gastrointestinal diseases, and/or other immune dysfunction diseases. Thus, patients who had previously received the aforementioned treatments or who were afflicted with the aforementioned comorbidities were excluded from the study (see Table S1 in the supplemental material).

### Stool sample collection and DNA extraction.

Fresh stool samples (0.25 g/sample) were collected in the morning from all study enrollees at the beginning of TB treatment and were stored at −80°C. Metagenomic DNA was extracted from each 0.25-g stool sample using a QIAamp PowerFecal Pro DNA kit (Qiagen, Germany) according to the manufacturer’s instructions. A Nanodrop 2000c spectrophotometer (ThermoFisher Scientific, USA) was used to measure DNA concentrations of extracted stool sample genomic DNA preparations, followed by storage of genomic DNA samples at −20°C.

### 16S rRNA gene sequencing.

Genomic DNA samples were sent to Berry Genomics Co., Ltd., for 16S rRNA gene sequencing and analysis. Briefly, conserved regions (V3 + V4) of rRNA genes were amplified with specific primers that contained barcode sequences. PCR products were recovered from gels and then quantified using a QuantiFluor fluorometer. Thereafter, a sequencing library was constructed, and the Illumina PE250 platform was then used for sequencing. Primer sequences were as follows: forward, 5′-GTGCCAGCMGCCGCGGTAA-3′; reverse, 5′-GGACTACHVGGGTATCTAAT-3′. Thirty-one stool samples were sequenced to obtain raw tag sequences, and the total tags were then spliced and filtered to obtain clean tags. Next, sequences based on clean tags were clustered into operational taxonomic units (OTUs) based on a 97% minimum identity cutoff. According to clustering results for OTUs, species annotation analysis was carried out using the Mothur method and the SILVA rRNA database (https://www.arb-silva.de) (38). We then analyzed gut microbiota compositions and abundance distributions for each sample at each taxonomic level (kingdom, phylum, class, order, family, and genus). Next, the Welch's *t* test was used to analyze differences between pairs of groups after exclusion of species with total abundance rates across all samples of <0.1%. Thereafter, α-diversity data, Venn plots, and petal plots of OTUs were analyzed to identify common and unique OTUs among different samples or groups. In addition, QIIME 2 software (version 2021.4) was used to calculate UniFrac distances for use in constructing an unweighted pair-group method arithmetic mean (UPGMA)-based sample cluster tree. PCA (principal-component analysis), PCoA (principal-coordinate analysis), and NMDS (nonmetric multidimensional scaling) diagrams were drawn using R software (version 2.15.3). R language was used to assess the statistical significance of differences between data sets by PERMANOVA using the weighted PCoA scores, and the significance of intergroup species differences was assessed using a *t* test and rank sum tests. Meanwhile, sequence data obtained via 16S rRNA gene sequencing of DNA obtained from human stool samples were stored in the China National GenBank Sequence Archive (CNSA) (39) of the China National GeneBank DataBase (CNGBdb) (40) under accession number CNP0001749/sub020106.

### ELISA.

Fresh whole blood samples were collected and centrifuged at 3,000 rpm/min for 10 min, and then plasma was collected. Plasma concentrations of human TNF-α, IL-1β, IL-6, IL-10, IL-12, and gamma interferon (IFN-γ) were then quantified using a human enzyme-linked immunosorbent assay (ELISA) kit (R&D Systems, MN, USA) according to the manufacturer’s instructions. All experiments were performed in triplicate. Mouse plasma cytokines were detected with a mouse plasma cytokine detection kit (R&D Systems) using the above-mentioned method.

### Isolation and identification of gut bacterial strains.

Fresh feces from patients with TBM were collected and smeared onto agar surfaces of MacConkey agar plates (Antu Biological Technology Co., Ltd., Zhengzhou, China), and then the plates were placed in a 37°C incubator for 18 to 24 h. Single colonies were then collected and separately cultured in Luria broth (LB) until the optical density at 600 nm (OD_600_) of each culture was ~0.6 to 0.8. Thereafter, the bacteria were harvested, and genomic DNA was extracted using the SDS method (41). Whole genomes of strains obtained from clinical TBM cases were sequenced by Beijing Novogene Bioinformatics Technology Co., Ltd. Agarose gel electrophoresis was used to assess DNA quality, and then genomic DNA samples were quantified using a Qubit 2.0 fluorometer (ThermoFisher Scientific). At least 1 μg of each DNA sample was subjected to ultrasonic fragmentation to generate DNA fragments of approximately 350 bp in size, and then DNA fragments were end polished and ligated to full-length adaptors, followed by additional PCR amplification and Illumina sequencing. Thereafter, the final PCR products were purified using an AMPure XP system (Beckman Coulter), and then the size distribution of the library was analyzed using an Agilent 2100 bioanalyzer. Quantification of library DNA was conducted via real-time PCR, and whole-genome sequencing was performed using the Illumina NovaSeq PE150 platform. Raw data were filtered and assembled using SOAP followed by SPAdes, AbySS, CISA, and then GapCloser to obtain the final assemblies. The GeneMarkS (42) program was used to predict protein coding sequences in the assemblies, and the nr (nonredundant) protein sequence database (43) was used to conduct coding sequence annotation. Serotyping of specific O-antigens for O typing and flagellin genes for H typing was performed using the SerotypeFinder CGE tool at the Center for Genomic Epidemiology (CGE) (44). Assembly data for the entire genome of each strain were stored in the CNSA of CNGBdb under accession number CNP0001749/sub020091.

### Bacterial culture and enumeration.

The clinical Escherichia coli isolate and M. tuberculosis strain H37Rv (ATCC 27294) were maintained by our laboratory. To generate the mouse gut microbiota dysbiosis model, E. coli was thawed and cultured in LB at 37°C until the OD_600_ reading of each culture reached 0.6, which was followed by CFU determination. For infection of mice with M. tuberculosis, a vial of M. tuberculosis H37Rv was thawed and cultured in Lowenstein-Jensen (L-J) medium for ~3 to 4 weeks. M. tuberculosis was then recovered, and cultures were adjusted to an OD_600_ of 0.6, followed by CFU determinations. For CFU determinations for E. coli strains, bacteria were serially diluted and cultured on LB agar at 37°C for 16 h, and colonies were counted to quantify bacterial loads. For CFU determinations of the M. tuberculosis strain, bacteria were diluted in 0.9% NaCl and spread onto surfaces of plates containing Middlebrook 7H10 agar base supplemented with 10% oleic acid-albumin-dextrose-catalase (OADC), followed by incubation of plates at 37°C for ~3 to 4 weeks. Thereafter, colonies were counted to quantify bacterial load per milliliter.

### Animal experiments.

Female C57BL/6 mice were purchased from Beijing Vitong Lihua Laboratory Animal Technology Co., Ltd. Mice were used in experiments after ~8 to 12 weeks of age. All mice were maintained on a strict 24-h reverse light-dark cycle (lights on from 10 p.m. to 10 a.m.) and allowed free access to food and water. For plasma cytokine level evaluations and collection of fecal samples for 16S rRNA gene sequencing, mice were randomly assigned to two groups: (i) a control group administered PBS by gavage (*n *= 6), and (ii) a study group administered a single E. coli clinical isolate by gavage from a fecal sample obtained from a TBM patient (*n *= 6). For E. coli colonization of mice, mice were administered 200 μL of bacteria suspended in PBS (5 × 10^9^ CFU) by gavage once a day each morning for 2 weeks. During the gavage period, food intake and body weights were recorded. Mouse blood samples were collected via retroorbital phlebotomy. Thereafter, the mice were deeply anesthetized, blood samples were collected to determine cytokine expression levels, and brain tissues were used to calculate tight junction protein expression.

For determinations of bacterial loads in brain tissues of mice infected with M. tuberculosis, 12 mice were randomly assigned to two groups: one group was administered PBS by gavage (*n *= 6) and one group was administered an E. coli clinical isolate by gavage (*n *= 6), with all gavage treatments administered each morning for 2 weeks. Each mouse was then administered 50 μL of M. tuberculosis H37Rv (1 × 10^6^ CFU) via intravenous tail vein injection, mortality and food intake were recorded, and body weights were measured last for 2 weeks. Thereafter, blood samples were taken from the tail vein of each animal for culture purpose at 1 day after infection. CFU counts of the blood were used to compare the infection efficacies between the two groups (45). In addition, the mice were deeply anesthetized and dissected to obtain brain specimens. The brain specimen of each mouse was divided into four parts: one for RNA extraction, one for protein extraction, one for mycobacterial enumeration, and one for histologic examination.

### RNA extraction and RT-qPCR.

Total RNA was extracted from left anterior brain tissues of mice using TRIzol reagent (Ambion, North America, USA) per the manufacturer’s instructions. After the concentration of each RNA preparation was determined using a spectrophotometer, 1 μg of RNA was reverse transcribed using cDNA Synthesis SuperMix (Yeasen Biotech Co., Ltd., Shanghai, China) per the manufacturer’s instructions. Next, claudin-5, occludin, and zonula occludens-1 (ZO-1) mRNA expression levels were determined via real-time quantitative PCR (RT-qPCR) using qPCR SYBR green master mix (Yeasen Biotech). PCR primers were synthesized by Tsingke Biotechnology Co. with the following names and sequences: mouse claudin-5 (forward, 5′-TTTCTTCTATGCGCAGTTGG-3′; reverse, 5′-GCAGTTTGGTGCCTACTTCA-3′); mouse occludin (forward, 5′-CCTTCTGCTTCATCGCTTCCTTA-3′; reverse, 5′-CGTCGGGTTCACTCCCATTAT-3′); mouse ZO-1 (forward, 5′-GATAGTTTGGCAGCAAGAGATGGTA-3′; reverse, 5′-AGGTCAGGGACGTTCAGTAAGGTAG-3′); and mouse glyceraldehyde-3-phosphate dehydrogenase (GAPDH) (forward, 5′-CCTCAACTACATGGTCTACA-3′; reverse, 5′-CCTGGAAGATGGTGATGG-3′). For mRNA analysis, reverse transcription was performed on total RNA using random primers (Promega). GAPDH was used as an internal control. Threshold cycle (*C_T_*) values were converted to relative fold changes of expression using the formula 2^−Δ^*^CT^*.

### Western blotting.

Right anterior mouse brain tissues were suspended in radioimmunoprecipitation assay (RIPA) buffer (Solarbio Life Sciences, Beijing, China) containing 1 mM phenylmethylsulfonyl fluoride (PMSF) (Solarbio Life Sciences), and then suspensions were homogenized to generate lysates using a FastPrep-24 5G instrument (MP Biomedicals, Santa Ana, CA, USA). After incubation for 30 min on ice, mixtures were centrifuged at 12,000 rpm for 10 min at 4°C, and then protein concentrations in the supernatants were determined using a bicinchoninic acid (BCA) protein assay kit (Solarbio Life Sciences). Next, 50 μg of protein in loading buffer for each sample was heated for 10 min at 98°C, and then proteins were separated via electrophoresis using 4% to 12% SDS-PAGE gels, followed by transfer of proteins to polyvinylidene difluoride (PVDF) membranes. After the membranes were blocked by immersion in 5% dried milk (wt/vol) in Tris-buffered saline with 0.1% Tween 20 (TBST) for 2 h at room temperature, they were probed with rabbit anti-claudin-5 polyclonal antibody (1:500, catalog no. 34-1600; Invitrogen) and mouse anti-β-actin antibody (1:5,000, catalog no. A2228; Sigma). Next, the membranes were washed and then incubated with the appropriate horseradish peroxidase (HRP)-conjugated secondary antibodies for 1 h at room temperature. Antibody-bound proteins on membranes were detected using SuperSignal west pico chemiluminescent substrate (Thermo Fisher Scientific, USA), and then the developed blots were imaged using X-ray film.

### Mycobacterial enumerations.

Mycobacterial enumerations were performed on mouse left posterior brain tissues. Brain specimens were homogenized in 5 volumes of 0.9% NaCl using a FastPrep-24 5G instrument (MP Biomedicals). Next, 100 μL of serial dilutions of brain homogenate was smeared onto the surfaces of plates containing Middlebrook 7H10 agar base supplemented with 10% OADC, the plates were incubated at 37°C for ~3 to 4 weeks, and the CFU were counted to evaluate brain tissue bacterial loads.

### ZN staining.

Acid-fast bacilli were detected using a commercial Ziehl-Neelsen (ZN) stain kit (Baso Diagnostics, Inc., China). Briefly, the 5-μm-thick formalin-fixed and paraffin-embedded right posterior brain tissue sections were deparaffinized and rehydrated through graded alcohol to distilled water. The sections were then flooded with carbolfuchsin for 10 min at room temperature. After the sections were washed in tap water for 2 min, acid alcohol was used for differentiation for 1 min. The sections were washed in tap water to remove excessive acid, and methylene blue was used for staining for 30 s. After the dehydrate was clear, the sections were mounted with neutral gum. Three hundred fields of each slide were examined by Nikon Eclipse 80i microscopy (Nikon, Japan) under oil immersion at a magnification of ×1,000.

### H&E staining.

Histological examination was performed on 5-μm-thick formalin-fixed and paraffin-embedded right posterior brain tissue sections using a hematoxylin-eosin (H&E) stain kit (Solarbio Life Sciences) according to the manufacturer’s instructions. Briefly, tissue sections were dewaxed and then hydrated. Next, sections were stained with hematoxylin solution for 10 min and then immersed in differentiation solution for 3 min. Thereafter, sections were stained with eosin by immersion in eosin Y aqueous solution, and then the stained sections were dehydrated by stepwise immersion in alcohol of increasing concentrations (75%, 85%, 95%, 100%). Finally, sections were cleared by immersion in xylene and then were sealed with neutral balsam (Solarbio Life Sciences, Beijing, China) prior to microscopic observation. Image capture was conducted using a Cytation 5 cell imaging multimode reader (BioTek, USA).

### Statistical analysis.

Data analysis was performed using IBM SPSS 25. For data that were normally distributed, continuous variables are represented as the mean ± standard error of mean (*x*¯  ± SEM). For data that were not normally distributed, the median and interquartile range (50th percentile, 25th percentile, 75th percentile) were used. Welch's *t* test or a nonparametric test was used to compare continuous variable data between pairs of groups, and one-way analysis of variance (ANOVA) with multiple comparisons was used for comparison of multiple groups. Categorical variables were compared using Pearson’s chi-square test or Fisher’s exact test as appropriate. Results with a *P *of < 0.05 were deemed statistically significant.

### Data availability.

The data for 16S rRNA gene sequences of human stool DNA have been stored in the CNGB Sequence Archive (CNSA) of the China National GeneBank DataBase (CNGBdb) under accession number CNP0001749/sub020106. Assembly data for the whole-genome sequencing of isolated gut microbial strains were stored in the CNSA of CNGBdb under accession number CNP0001749/sub020091.
