# Supplementary material for: Predominance of Escherichia-Shigella in Gut Microbiome and Its Potential Correlation with Elevated Level of Plasma Tumor Necrosis Factor Alpha in Patients with Tuberculous Meningitis
Source: Microbiol Spectr. 2022 Nov 9;10(6):e01926-22. doi: 10.1128/spectrum.01926-22 (PMC9769903; doi:10.1128/spectrum.01926-22)
Supplement: Supplemental file 1 — Supplemental material. Download spectrum.01926-22-s0001.pdf, PDF file, 0.6 MB [file spectrum.01926-22-s0001.pdf]

Supplementary Material

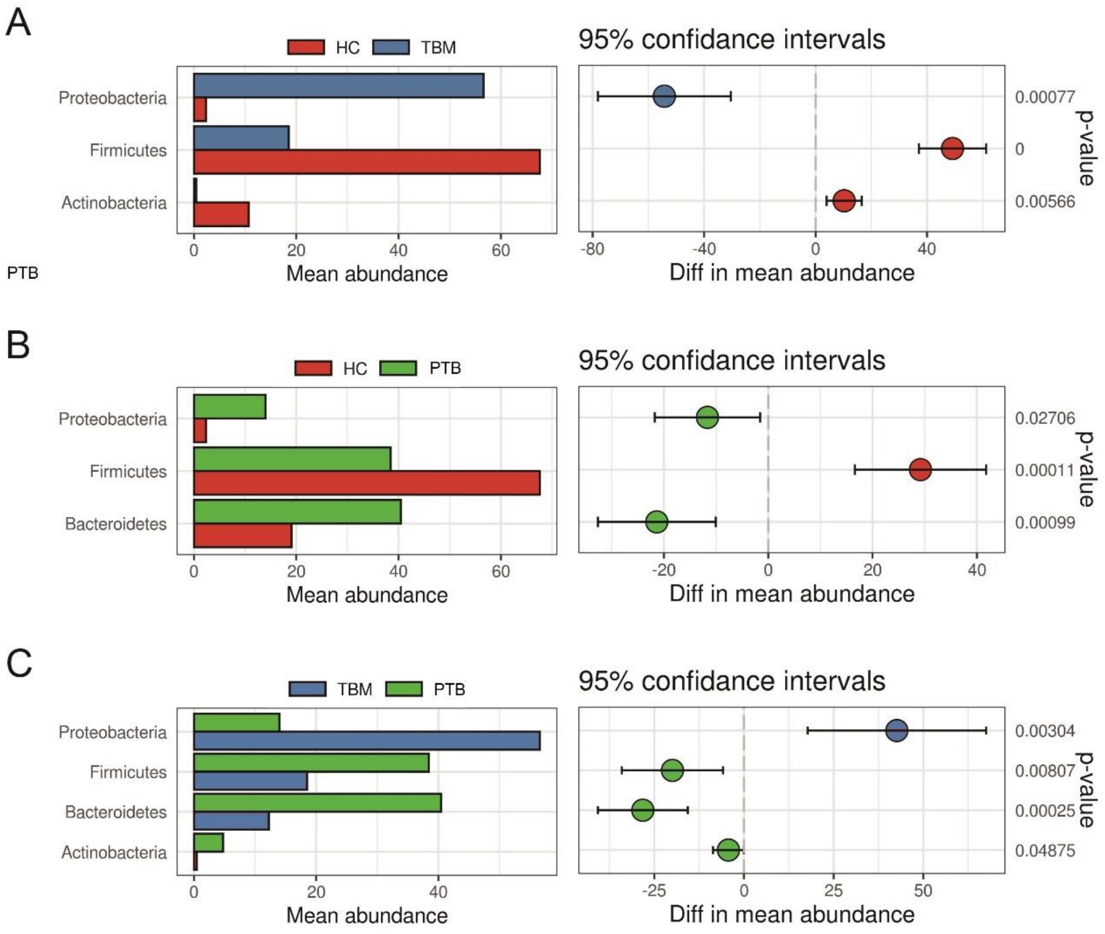

Supplementary Figure. 1 Comparisons were made between HC, PTB, and TBM microbiota compositions at the phylum level.

Welch's t-test was used to analyze phylum-level differences in microbiota compositions between pairs of groups: HC and TBM (A), HC and PTB (B), TBM and PTB (C). Phyla with total abundance rates of <0.1% were filtered out, as were results with p-values below the threshold of <0.05. HC: healthy control; PTB: patients with pulmonary tuberculosis; TBM: patients with tuberculosis meningitis.

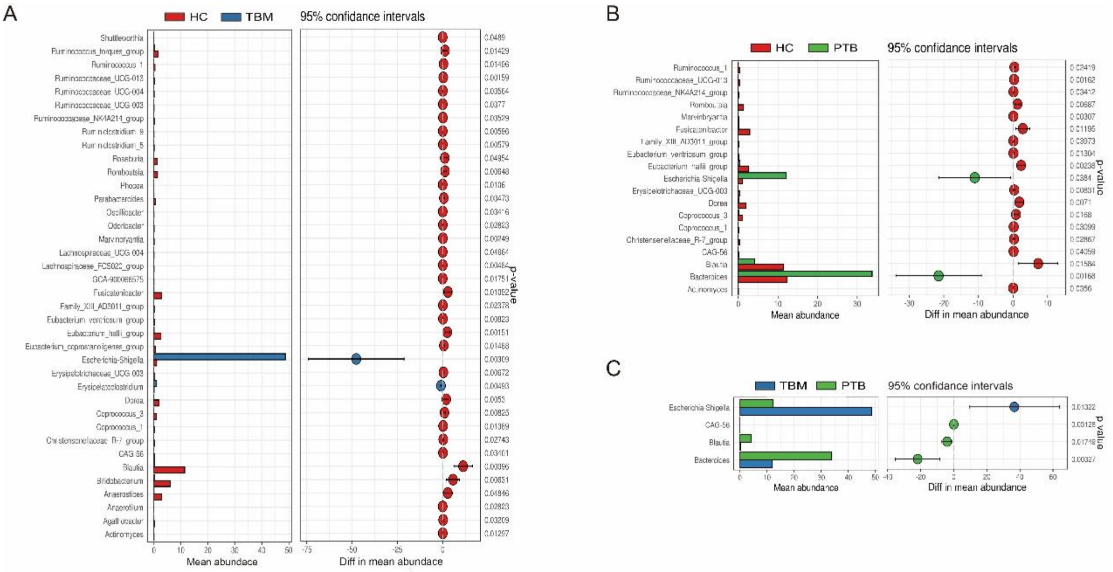

Supplementary Figure. 2 Comparisons were made between HC, PTB, and TBM microbiota compositions at the genus level.

Welch' s t-test was used to analyze genus-level differences in microbiota compositions between pairs of groups: TBM and HC (A), PTB and HC (B), TBM and PTB (C) group. Phyla with total abundance rates of <0.1% were filtered out, as were results with p-values below the threshold of <0.05. HC: healthy control; PTB: patients with pulmonary tuberculosis; TBM: patients with tuberculosis meningitis.

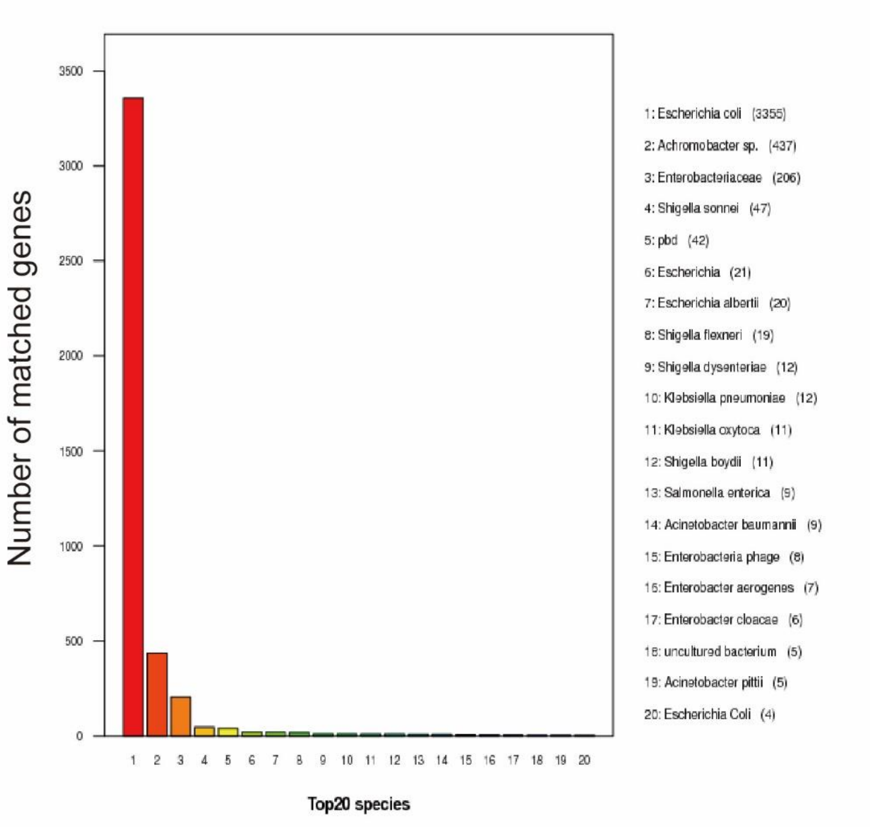

Supplementary Figure.3 Identification of Dominant Intestinal Species of Escherichia-Shigella in TBM patients

Results of whole-genome sequencing-derived protein coding sequences searched against the nr protein database

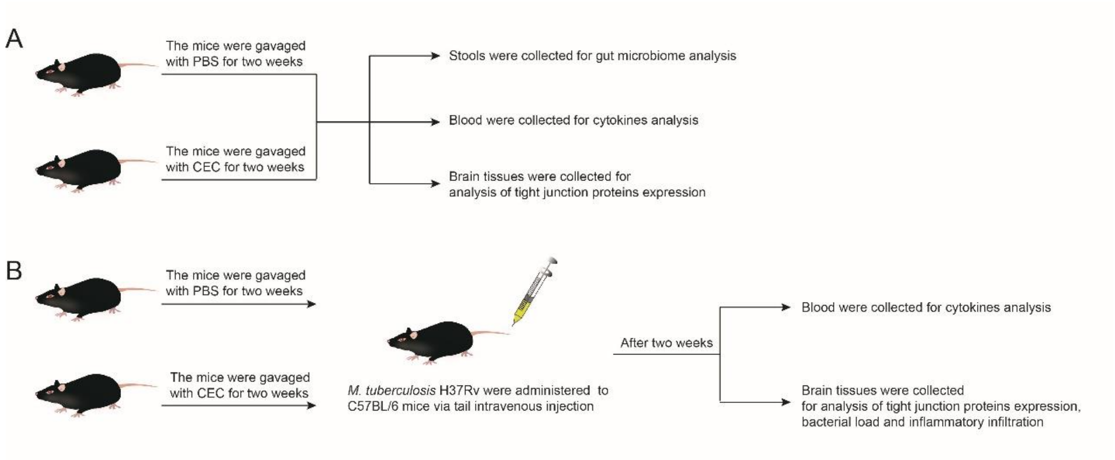

Supplementary Figure.4 Experimental design flow chart of gut microbiome dysbiosis animal model

(A) Groups of six C57BL/6 mice were gavaged daily for 2 weeks with CEC isolate or PBS (control). Then, the mice were euthanatized and stools, blood, and brain tissues were collected for analysis gut microbiome, cytokine levels, and tight junction protein expression; (B) Groups of six C57BL/6 mice were gavaged daily for 2 weeks with CEC isolate or PBS (control). Thereafter, mice were injected with 1×10^6 colony forming units (CFUs) of *M. tuberculosis*. At 2 weeks post-infection, the mice were euthanatized and blood and brain tissues of mice were collected for analysis of cytokine levels, tight junction protein expression, bacterial load, and inflammatory infiltration. CEC: clinical *E. coli* isolate.

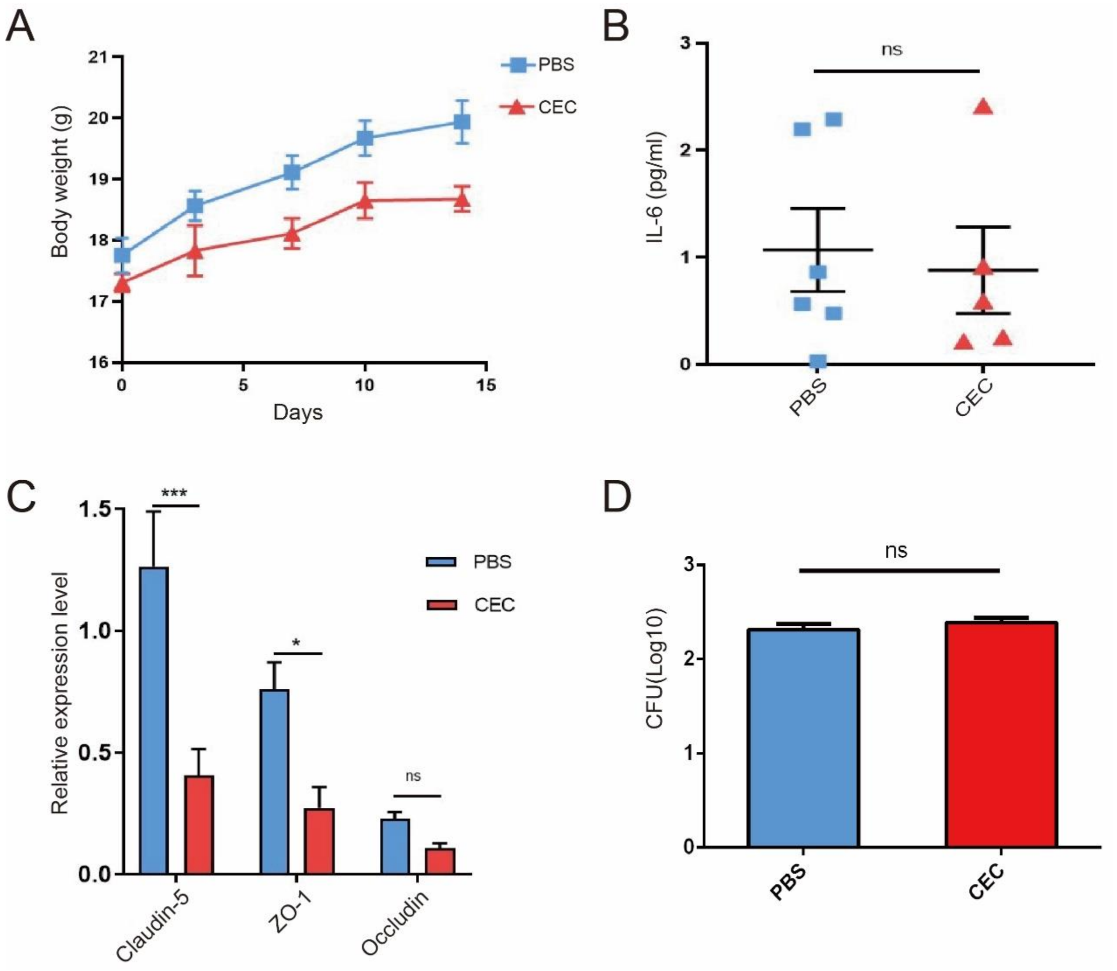

Supplementary Figure.5 The murine model with gut microbiota dysbiosis was constructed via CEC gavage.

(A) Body weight curves of mice in the two groups after gavage with PBS or CEC; (B) IL-6 levels in serum from C57BL/6 mice gavaged with PBS or CEC; (C) Quantitative analysis of claudin-5, ZO-1, and occludin mRNA expression levels in brains from C57BL/6 mice gavaged with PBS or CEC. (D) Blood bacterial counts 1 day after *M. tuberculous* injection of mice previously treated via gavage with CEC or PBS. Each result is shown as the mean ± SEM as determined based on analysis conducted using the Kruskal-Wallis test (ns. denotes not significant, ****P* < 0.001, ***P* < 0.01, **P* < 0.05. CEC: clinical *E. coli* isolate).

Table S1. Inclusion/exclusion criteria of individuals in this study

a) Inclusion criteria

Cohort	Inclusion criteria
HC	(1) aged 18-70;<br>(2) healthy BMI;<br>(3) normal liver and kidney function, normal stools, and normal levels of fasting blood glucose, blood lipids, and urinary function indicators;<br>(4) no history of pulmonary and brain diseases;<br>(5) negative interferon gamma release assay (IGRA) results.
PTB	(1) aged 18-70;<br>(2) positive M. tuberculosis detection results obtained for sputum smears and/or cultures and/or positive GeneXpert MTB/RIF results (Cepheid, USA);<br>(3) pulmonary imaging features indicative of TB.
TBM	(1) aged 18-70;<br>(2) tested positive for *M. tuberculosis* complex bacilli in cerebrospinal fluid (CSF) by GeneXpert MTB/RIF assay.

b) Exclusion criteria

Exclusion criteria
(1) prior treatment with systemic anti-tuberculous therapy for more than one week;
(2) received treatment with extensive antibiotic therapy for more than 1 week during the previous 6 months;
(3) prior treatment with any steroid drugs;
(4) comorbidities, such as diabetes, malignancy, gastrointestinal diseases, and/or other immune dysfunction diseases.

[a]HC: health control; [b]BMI: body mass index; [c]PTB: patients with pulmonary tuberculosis; [d]TBM: patients with tuberculosis meningitis.

Table S2: PerMANOVA test of the differences in microbiome composition

Groups	*P* values
HC *vs.* PTB	0.001
HC *vs.* TBM	0.001
PTB *vs.* TBM	0.001
HC *vs.* PTB *vs.* TBM	0.001

HC: healthy control; PTB: patients with pulmonary tuberculosis; TBM: patients with tuberculosis meningitis. $P < 0.05$ was deemed statistically significant.
